# Heritability and genetic and environmental correlations of heart rate variability and baroreceptor reflex sensitivity with ambulatory and beat-to-beat blood pressure

**DOI:** 10.1038/s41598-018-38324-6

**Published:** 2019-02-07

**Authors:** Tengfei Man, Harriëtte Riese, Deepali Jaju, M. Loretto Muñoz, Mohammed O. Hassan, Said Al-Yahyaee, Riad A. Bayoumi, Anthony G. Comuzzie, John S. Floras, Arie M. van Roon, Ilja M. Nolte, Sulayma Albarwani, Harold Snieder

**Affiliations:** 1 Department of Epidemiology, University of Groningen, University Medical Center Groningen, Groningen, The Netherlands; 2 Interdisciplinary Center Psychopathology and Emotion regulation, Department of Psychiatry, University of Groningen, University Medical Center Groningen, Groningen, The Netherlands; 3 College of Medicine and Health Sciences, Sultan Qaboos University, Muscat, Sultanate of Oman; 4 Canadian Health Centre, Muscat, Sultanate of Oman; 5 Department of Biochemistry, College of Medicine and Health Sciences, Sultan Qaboos University, Muscat, 123 Sultanate of Oman; 6 College of Medicine, Mohammed Bin Rashid University for Medicine and Health Science, Dubai, UAE; 7 Department of Genetics, Texas Biomedical Research Institute, Texas, USA; 8 University Health Network and Mount Sinai Hospital Division of Cardiology, Department of Medicine, University of Toronto, Toronto, Ontario, Canada; 9 Department of Vascular Medicine, University of Groningen, University Medical Center Groningen, Groningen, The Netherlands

## Abstract

This family study from Oman (n = 1231) explored the heritability and genetic and environmental correlations of heart rate variability (HRV) and baroreceptor reflex sensitivity (BRS) with ambulatory and beat-to-beat blood pressure (BP). Ambulatory BP was measured for 24 hours to calculate mean values for daytime and sleep separately. Time and frequency domain HRV indices, BRS, office beat-to-beat BP, and heart rate (HR) were measured for 10 minutes at rest. SOLAR software was used to perform univariate and bivariate quantitative genetic analyses adjusting for age, age^2^, sex, their interactions and BMI. Heritability of SBP and DBP ranged from 16.8% to 40.4% for daytime, sleeping, 24-hour and office beat-to-beat measurements. HR and BRS showed a heritability of 31.9% and 20.6%, respectively, and for HRV indices heritability ranged from 11.1% to 20.5%. All HRV measurements and BRS were found to be negatively correlated with BP, but phenotypic correlation coefficients were relatively weak; HR was positively correlated with BP. None of the genetic correlations were statistically significant while environmental factors explained most of the correlations for all HRV indices with BP. Our study found consistent but weak correlations among HRV, HR, BRS and ambulatory/office beat-to-beat BP. However, environmental rather than genetic factors contributed most to those correlations.

## Introduction

Hypertension (HTN) is a progressive cardiovascular syndrome and is usually defined by the presence of a chronic elevation of systemic arterial pressure above a certain threshold value. Blood pressure (BP) serves as the underlying biomarker that defines HTN and it is commonly used as key indicator to assess the disease severity combined with other CV risk factors^1^. Two common methods are typically used to measure BP at the upper arm for diagnosis of HTN: traditional BP measurement in the physician’s office and ambulatory BP measurement^2^.

Higher BP or HTN has been found to be associated with higher heart rate (HR) and lower heart rate variability (HRV) in cross-sectional^3,4^ and prospective studies^5,6^. HRV is the physiological phenomenon of variation in the time interval between heartbeats and is a quantitative marker of the autonomic modulation of sinus node discharge^7^. The baroreflex feedback loop is an important cardiovascular control mechanism for short-term BP regulation as it registers and dampens fluctuations in BP through sympathetic and parasympathetic nervous system modulation of, especially, the HR. Baroreceptor reflex sensitivity (BRS) can be defined as the transfer function between BP and HR changes^8^. A reduction in BRS has been shown to lead to a reduced buffering of BP fluctuations^9^, which in time can potentially result in a higher absolute BP setpoint around which the BP level is regulated^10^.

Previous twin and family studies indicated that BP, HR, HRV and BRS are all heritable (genetic factors contributing a substantial part of the variance in those traits). A systematic review and meta-regression of twin studies published recently reported that the mean (95% CI) heritabilities for systolic BP, diastolic BP and HR were 0.54 (0.48–0.60), 0.49 (0.42–0.56), and 0.61 (0.51–0.70), respectively^11^. A recent review on the genetics of autonomic nervous system activity summarized the results from previous twin studies with a sample size over 50 twin pairs and reported heritability estimates between 25 and 70% for the different indices of HRV and between 22 and 55% for BRS^12^.

Given that both BP and indices of autonomic nervous system activity (HRV, BRS) are heritable raises the question whether the inverse associations between these variables identified in observational studies may partly be explained by shared genetic factors influencing both classes of phenotypes. This hypothesis has not been explored previously. Population based family study designs have the potential to facilitate the analysis of the effects of both genes and environment. We will, therefore, test this hypothesis with data from the Oman Family Study (OFS), an isolated population that is environmentally and genetically homogeneous^13^. In the OFS, ambulatory BP (daytime, sleeping, and 24-hour) and office beat-to-beat BP were collected together with laboratory assessments of a wide range of HRV indices, HR and BRS in a large number of participants.

## Methods

### Study Population

Five large, extended and highly consanguineous families (1231 subjects in total and 304, 142, 225, 279 and 281 persons separately), each living in a separate village within a perimeter of 20 km around the City of Nizwa were selected. Interviewed people represented approximately 10–15% of the total number of individuals in these five pedigrees. They were 16–80 years old and all voluntarily took part in the study, appeared healthy, and had no clinical complaints as determined by a questionnaire. In these five pedigrees, first cousin marriages represent >50% of all marriages. Polygamy is widely practiced with some men marrying up to 4 wives^14^. The study was approved by the Medical Research and Ethics Committee of Sultan Qaboos University. A written and signed or thumb-printed rubber-stamped informed consent was obtained from each subject or a parent and/or legal guardian if subjects were under the age of 18 years.

### Ambulatory BP Measurements

Ambulatory BP measurements were recorded during the course of a whole 24-hour day on a first home visit, using the auscultatory mode of the validated Schiller BR 102 ambulatory BP monitor (Baar, Switzerland). With the subject seated the appropriate size cuff was fixed to the non-dominant arm and three BP readings taken. During the same home visit three BP readings were also taken with a calibrated mercury sphygmomanometer on the dominant arm to confirm accuracy of the ambulatory BP measurements. Recordings were accepted and ambulatory BP recordings started when the average of both measurement methods did not differ by >5 mmHg. To reduce movement artefacts during ambulatory BP recordings, subjects were discouraged from strenuous physical activity. The BP monitor was programmed to record BP every 30 minutes from 07:30 to 21:30 and every 60 minutes from 21:30 to 09:30, for a total of 26 hours. The first 2 hours of monitoring were considered as an adaptation period and were not included in the calculation of BP means. Recordings were accepted when the rate of invalid measurements due to e.g. artefacts was <25% and when the recording lasted for at least 20 consecutive hours. Quality control of data output from the 24-hour monitor for SBP and DBP was performed by one technician, trained at identifying artefacts and outliers. The daytime and sleep periods were determined for each subject according to their actual waking and sleeping time as recorded in their diaries and confirmed by changes in BP. The average BP levels during the total 24 hours and during daytime and sleep periods were calculated^15^.

### Beat-to-beat BP and HR/HRV measurement

After reporting to the field research center at 7.00 am and removing the BP monitor subjects were made to rest in supine position for 10 minutes on a comfortable bed, in a quiet office with a temperature between 24 and 26 °C. Measurements were acquired for the subsequent 10 minutes using the Task Force Monitor (CNS systems, Graz, Austria). The beat-to-beat BP was recorded using the vascular unloading technique whereby finger cuff readings were recorded, automatically counterchecked and corrected every minute, by the oscillometric BP measurements recorded from the contralateral upper arm^15^. Subjects (n = 10) with more than 5% of their ECG signals not meeting our ECG signal criteria (i.e. NN-intervals between 300 ms and 2000 ms and successive NN-interval ratios between 0.8 and 1.2) were excluded. Measurements (n = 3) that deviated by more than 4 standard deviations (SD) from the mean for a trait, were set to missing for the corresponding trait. Subjects taking anti-hypertensives (n = 189) were not asked to stop medication, but the measured BP results were corrected (+15 mmHg for SBP and +10 mmHg for DBP) prior to analysis^16,17^.

Inter beat intervals (IBI, or RR-intervals) were obtained from lead II of a 6-lead ECG and used to calculate average HR and HRV. Time domain HRV indices included the standard deviation of the normal-to-normal interval (SDNN) and the square root of the mean squared differences of successive normal-to-normal intervals (RMSSD). Frequency domain measures were high frequency (HF, 0.15–0.40 Hz; reflecting the strength of HR modulation by the parasympathetic system, i.e., the vagus nerve), low frequency (LF, 0.04–0.15 Hz; reflecting a mixture of sympathetic and parasympathetic activity) and very low frequency (VLF, 0.003–0.15 Hz; reflecting a host of factors, including not only the sympathetic nervous system, but also input from chemoreceptors, thermoreceptors, the renin-angiotensin system and others) and the sum of these frequency bands (HF + LF + VLF) giving an estimate of the total power (TP, 0–0.4 Hz, reflecting the overall autonomic activity). All these are commonly used HRV indices^7,18^

### BRS Calculation

BRS was calculated based on the computer identification of spontaneously occurring sequences of four or more beats in the time domain and slope of the regression line between SBP and RR interval changes is taken as an index of the BRS. The sequence should have consecutive four or more beats showing an increase in SBP associated with prolongation of RR-interval (up-up events) or a fall in SBP with shortening RR-interval (down-down events). Since these sequences are spontaneous, only those with a consecutive 1 mmHg change in SBP and at least 1 ms change in R-R interval were included into the analysis. Consequently, there is a high proportion of missing data for the BRS based on up-up events (31.3%) and down-down events (28.4%). To optimize sample size based on all available data of up-up and/or down-down events we calculated a new average BRS variable [BRS = (BRS up-up + BRS down-down)/2] if both of them were available and used only BRS up-up or BRS down-down if either of them was missing. Distributional characteristics of the log-transformed BRS up-up (mean [SD] = 2.61 [0.69]) and log-transformed BRS down-down (mean [SD] = 2.62 [0.67]) variables were highly similar (distribution of log-transformed BRS up-up and down-down variables is shown in Supplemental Fig. 1).

### Statistical Analysis

Descriptive statistics were used to present the baseline characteristics of the study sample and Student’s t-tests were used to test for sex differences in the means. Prior to analysis, distributions of all variables were checked. To obtain better approximations of normal distributions of HR, HRV and BRS, measurements were transformed by natural logarithm (distributions of these variables before and after log transformation are shown in Supplemental Fig. 2). SOLAR (v7.2.5), a standard software package for variance components and linkage analysis of family data was used to perform univariate and bivariate analyses^19,20^.

1 $${{\rm{\sigma }}}_{{\rm{P}}}^{2}=2{{\rm{\Phi }}{\rm{\sigma }}}_{{\rm{G}}}^{2}+{I{\rm{\sigma }}}_{{\rm{E}}}^{2}$$

SOLAR uses a variance-component method to analyze family-based quantitative data by decomposing the overall phenotypic variance into genetic and environmental components using the observed covariance in the trait among family members (Φ is n*n matrix of kinship coefficients that structures σ^2^_G_ i.e. the variance due to the additive genetic effects [σ^2^_G_]; and I is the identity matrix of order n). Each genetic and environmental variance component is accompanied by a structuring matrix that predicts the covariance among individuals associated to that component. The structuring matrix for σ^2^_G_ is twice the kinship coefficient (2Φ) and for unmeasured, non-genetic factors (i.e. σ^2^_E_) it is the identity matrix I, this is shown in equation 1.

2 $${{\rm{h}}}^{2}={{\rm{\sigma }}}_{{\rm{G}}}^{2}/{{\rm{\sigma }}}_{{\rm{P}}}^{2}$$

SOLAR estimates additive genetic or narrow sense heritability which is the proportion of the phenotypic variance (σ^2^_P_) attributed to additive genetic effects (σ^2^_G_) using equation 2. The h^2^ significance was determined by using a likelihood ratio test where the log-likelihood of the estimated model is compared to the nested model where σ^2^_G_ is fixed to zero^21^.

3 $${r}_{p}=(\sqrt{{h}_{BP}^{2}}\times {r}_{G}\times \sqrt{{h}_{HRV}^{2}})+(\sqrt{{e}_{BP}^{2}}\times {r}_{E}\times \sqrt{{e}_{HRV}^{2}})$$

In our study the bivariate quantitative genetic analyses were conducted to estimate the genetic and environmental correlations of HRV, HR and BRS with BP. This is an extension of equation 1 where the phenotypic covariance between two individuals for two traits is given by a 2*2 covariance matrix resulting in equation 3, where r_G_ is the additive genetic correlation and r_E_ is the environmental correlation between HRV and BP.

With the additive genetic correlation (r_G_) we measured the extent of common genetic effects on the two traits being analyzed (i.e. pleiotropy). To test for the significance of shared genetic effects (|r_G_| >0), r_G_ was first estimated and subsequently fixed to zero in a nested sub-model allowing for a comparison of the two models using a likelihood ratio test. Similarly to test for complete overlap of genetic effects (r_G_ = 1), r_G_ was fixed to one and compared to the more general model in which it was freely estimated. If r_G_ = 0, this means that two traits being analyzed are influenced by independent genetic factors. If r_G_ = 1 then the genetic factors are completely shared^21,22^.

Sex, age, BMI, age^2^, sex by age and sex by age^2^ interactions were included as covariates in the analyses. P-values < 0.05 were considered statistically significant. Age^2^ was included to take potential non-linear effects of age on outcome phenotypes into account. All study procedures were performed in accordance with the relevant guidelines and regulations.

## Results

A total of 1231 subjects with a median age of 28 years old (Interquartile Range, IQR: 21–45) were included in the final analysis. Slightly more women participated in the study (54.9%). Men were taller and heavier, but no significant sex differences were found for age and BMI. In general, men had significantly higher BP, HRV and BRS, but lower HR than women (Table 1).

**Table 1:** Descriptive statistics stratified by sex.

Characteristics	Men		Women		p-value
	N	Statistic^a^	N	Statistic^a^	
**Anthropometric measurement**					
Age (years)	554	26.5 (20.0–42.0)	677	30.0 (22.0–45.0)	NS
Height (cm)	554	166.0 (161–170.1)	677	152.0 (149.0–155.5)	<0.01
Weight (kg)	554	67.0 (58.0–77.0)	677	56.0 (49.5–67.0)	<0.01
Body mass index (kg/m^2^)	554	24.6 (20.9–28.2)	677	24.7 (21.2–28.6)	NS
**Ambulatory and office beat-to-beat BP**					
Daytime SBP (mmHg)	534	127.0 (121.0–135.0)	644	119.0 (111.0–128.0)	<0.01
Daytime DBP (mmHg)	534	82.0 (76.8–89.0)	644	80.0 (74.0–88.0)	<0.01
Sleep SBP (mmHg)	513	109.0 (102.0–118.5)	625	104.0 (95.0–114.5)	<0.01
Sleep DBP (mmHg)	513	68.0 (61.0–75.0)	625	66.0 (59.0–73.0)	<0.05
Total 24-hour SBP (mmHg)	523	124.0 (117.0–131.0)	632	116.0 (108.0–125.0)	<0.01
Total 24-hour DBP (mmHg)	523	79.0 (74.0–86.0)	632	77.0 (71.3–84.0)	<0.01
Office beat-to-beat SBP (mmHg)	507	116.4 (108.2–124.9)	616	105.1 (98.2–113.0)	<0.01
Office beat-to-beat DBP (mmHg)	507	74.0 (68.0–82.1)	616	66.6 (60.4–73.0)	<0.01
**Heart rate (variability) and Baroreceptor reflex sensitivity**					
SDNN (ms)	523	64.4 (47.8–81.4)	624	51.0 (37.6–65.5)	<0.01
RMSSD (ms)	523	39.5 (28.4–56.1)	624	33.9 (22.8–49.6)	<0.01
lnHF (ms^2^)	498	10.8 (1.4)	610	10.4 (1.5)	<0.01
lnLF (ms^2^)	500	4.2 (1.2)	610	3.4 (1.3)	<0.01
lnVLF (ms^2^)	500	3.4 (1.1)	610	2.8 (1.2)	<0.01
lnTP (ms^2^)	500	5.1 (1.1)	610	4.5 (1.2)	<0.01
HR (beats/min)	526	66.9 (61.5–74.1)	629	72.6 (66.0–79.2)	<0.01
lnBRS (ms/mmHg)	471	2.7 (0.6)	555	2.6 (0.7)	<0.01

SDNN: standard deviation of normal-to-normal intervals; BP: blood pressure; SBP: systolic blood pressure; DBP: diastolic blood pressure; RMSSD: root mean square of successive differences; HF: high frequency; LF: low frequency; VLF: very low frequency; TP: total power; HR: heart rate; BRS: baroreceptor sensitivity; SDNN, RMSSD and HR were transformed by natural logarithm prior to testing for sex differences. NS: not significant.

The daytime and sleep periods were determined for each subject according to their actual waking and sleeping time as recorded in their diaries.

a:Data expressed as mean (sd) for normally distributed variable or median [IQR] in case of skewed distribution.

Results of the univariate analyses showed that h^2^ of SBP and DBP vary among daytime, sleeping, total 24-hour, and office beat-to-beat measurements ranging from 16.8% to 40.4% (Table 2). HR and BRS showed a heritability of 31.9% and 20.6%, respectively, and HRV indices ranged from 11.1% to 20.5%. Heritability estimates of all the traits differed significantly from 0 (p < 0.01). Heritability estimation for BRS was similar (h^2^ = 19.2%) when only cases with complete data on both up-up and down-down BRS (n = 785) were considered.

**Table 2:** Heritability estimates of BP, BRS, HRV and HR.

Traits	N	h^2^	SE	*p-value*
**Ambulatory and office beat-to-beat BP**				
Daytime SBP	1178	0.302	0.048	<0.01
Daytime DBP	1178	0.382	0.052	<0.01
Sleep SBP	1138	0.214	0.049	<0.01
Sleep DBP	1138	0.168	0.047	<0.01
Total 24-hour SBP	1155	0.321	0.051	<0.01
Total 24-hour DBP	1155	0.404	0.054	<0.01
Office beat-to-beat SBP	1123	0.244	0.053	<0.01
Office beat-to-beat DBP	1123	0.220	0.052	<0.01
**Heart rate (variability) and Baroreceptor reflex sensitivity**				
lnSDNN	1147	0.123	0.048	<0.01
lnRMSSD	1147	0.205	0.059	<0.01
lnHF	1108	0.175	0.051	<0.01
lnLF	1110	0.137	0.045	<0.01
lnVLF	1110	0.111	0.039	<0.01
lnTP	1110	0.128	0.046	<0.01
lnHR	1155	0.319	0.060	<0.01
lnBRS	1026	0.206	0.058	<0.01

*Models were adjusted for age, sex, BMI, age^2^, age × sex, and age^2^ × sex.

*BP: blood pressure; SBP: systolic blood pressure; DBP: diastolic blood pressure; SDNN: standard deviation of normal-to-normal intervals; RMSSD: root mean square of successive differences; HF: high frequency; LF: low frequency; VLF: very low frequency; TP: total power; HR: heart rate; BRS: baroreceptor sensitivity. ln: indicates that data were ln-transformed prior analysis.

The results of the bivariate quantitative genetic analyses showed consistently weak (r: −0.18 to 0.001) phenotypic correlations of HRV measurements (including SDNN, RMSSD, HF, LF, VLF and TP), with BP (Tables 3–4). Significant phenotypic correlations were found for SDNN and LF with sleeping ambulatory SBP and for all HRV measures except VLF with sleeping ambulatory DBP, for RMSSD with both 24-hour ambulatory SBP and DBP and for TP with 24-hour DBP only, for SDNN, RMSSD and HF with both office beat-to-beat SBP and DBP and for TP with office beat-to-beat SBP only. None of the genetic correlations contributed significantly to the phenotypic correlations between HRV and ambulatory/office beat-to-beat BP, but some significant environmental correlations were found, especially for the phenotypic correlations between HRV and office beat-to-beat BP.

**Table 3:** Bivariate quantitative genetic analyses of HRV, HR, and BRS with ambulatory daytime/sleep/24-hour SBP/DBP examining the genetic, environmental, and phenotypic correlations.

	N	Ambulatory Daytime SBP				Ambulatory Daytime DBP			
		Genetic correlation, r_G_(SE)	Environmental correlation, r_E_(SE)	Phenotypic correlation, r_P_	Proportions of r_P_ (A/E)	Genetic correlation, r_G_(SE)	Environmental correlation, r_E_(SE)	Phenotypic correlation, r_P_	Proportions of r_P_ (A/E)
lnSDNN	1223	0.09 (0.17)	−0.10^*^ (0.05)	−0.06	0.72/0.28	0.04 (0.16)	−0.08(0.05)	−0.05	0.13/0.87
lnRMSSD	1223	0.02 (0.16)	−0.09(0.05)	−0.06	0.06/0.94	−0.03 (0.15)	−0.07(0.05)	−0.06	0.14/0.86
lnHF	1215	0.01 (0.16)	−0.07(0.05)	−0.05	0.04/0.96	−0.01 (0.15)	−0.06(0.05)	−0.04	0.06/0.94
lnLF	1215	0.08 (0.17)	−0.06(0.05)	−0.03	0.26/0.74	−0.07 (0.16)	−0.01(0.05)	−0.03	0.69/0.31
lnVLF	1215	0.15 (0.19)	−0.03(0.05)	0.001	0.54/0.46	−0.03 (0.18)	−0.01(0.05)	−0.01	0.45/0.55
lnTP	1215	0.06 (0.18)	−0.06(0.05)	−0.04	0.20/0.80	−0.06 (0.17)	−0.04(0.05)	−0.05	0.31/0.69
lnHR	1223	−0.14 (0.13)	0.07(0.05)	0.005	0.48/0.52	0.03 (0.13)	0.02(0.06)	0.02	0.45/0.55
lnBRS	1211	−0.17(0.15)	−0.06(0.05)	−0.09^§^	0.49/0.51	−0.11(0.15)	−0.07(0.06)	−0.08^§^	0.43/0.57
	**N**	**Ambulatory Sleep SBP**				**Ambulatory Sleep DBP**			
lnSDNN	1214	−0.02 (0.19)	−0.08(0.05)	−0.06^§^	0.05/0.95	−0.13 (0.21)	−0.07(0.05)	−0.10^§^	0.27/0.73
lnRMSSD	1214	0.09 (0.19)	−0.07(0.05)	−0.04	0.25/0.75	−0.08 (0.21)	−0.08^*^ (0.05)	−0.08^§^	0.18/0.82
lnHF	1204	0.08(0.19)	−0.07(0.05)	−0.04	0.23/0.77	0.03 (0.20)	−0.09(0.05)	−0.07^§^	0.07/0.93
lnLF	1205	0.05 (0.20)	−0.09(0.05)	−0.07^§^	0.11/0.89	0.01 (0.22)	−0.08 (0.05)	−0.07^§^	0.02/0.98
lnVLF	1205	0.15 (0.22)	−0.04(0.05)	−0.01	0.44/0.56	0.14 (0.24)	−0.08(0.05)	−0.05	0.22/0.78
lnTP	1205	0.10 (0.21)	−0.09(0.05)	−0.06	0.20/0.80	0.09 (0.23)	−0.11^*^ (0.05)	−0.18^§^	0.13/0.87
lnHR	1214	−0.05 (0.16)	0.07(0.05)	0.04	0.23/0.77	0.05 (0.17)	0.08(0.05)	0.07^§^	0.17/0.83
lnBRS	1197	−0.19(0.18)	−0.03(0.05)	−0.07^§^	0.67/0.33	−0.23(0.20)	−0.04(0.05)	−0.08^§^	0.58/0.42
	**N**	**Ambulatory 24-hour SBP**				**Ambulatory 24-hour DBP**			
lnSDNN	1216	0.08 (0.17)	−0.10(0.05)	−0.06	0.19/0.81	0.01 (0.16)	−0.08(0.05)	−0.05	0.04/0.96
lnRMSSD	1216	0.02 (0.16)	−0.10(0.05)	−0.07^§^	0.06/0.94	−0.04 (0.15)	−0.09(0.05)	−0.07^§^	0.15/0.85
lnHF	1206	0.02 (0.16)	−0.08(0.05)	−0.06	0.07/0.93	−0.04 (0.15)	−0.07(0.05)	−0.06	0.18/0.82
lnLF	1207	0.09 (0.17)	−0.08(0.05)	−0.04	0.24/0.76	−0.07 (0.16)	−0.04(0.05)	−0.04	0.37/0.63
lnVLF	1207	0.15 (0.19)	−0.05(0.05)	−0.01	0.43/0.57	−0.02 (0.18)	−0.03(0.05)	−0.02	0.16/0.84
lnTP	1207	0.07 (0.18)	−0.08(0.05)	−0.05	0.19/0.81	−0.06 (0.17)	−0.07(0.05)	−0.06^§^	0.22/0.78
lnHR	1216	−0.13 (0.13)	0.10(0.06)	0.02	0.38/0.62	0.02 (0.13)	0.06(0.06)	0.04	0.16/0.84
lnBRS	1201	−0.19(0.15)	−0.06(0.05)	−0.10^§^	0.53/0.47	−0.14(0.15)	−0.06(0.06)	−0.08^§^	0.50/0.50

Models were adjusted for age, sex, BMI, age^2^, age × sex, and age^2^ × sex.

SBP: systolic blood pressure; DBP: diastolic blood pressure; r_G:_ genetic correlation; r_E_: environmental correlation; r_P_: phenotypic correlation; SE: standard error; SDNN: standard deviation of normal-to-normal intervals; RMSSD: root mean square of successive differences; HF: high frequency; LF: low frequency; VLF: very low frequency; TP: total power; HR: heart rate; BRS: baroreceptor sensitivity.

^†^Genetic correlations are significantly different from 0 (p < 0.05).

*Environmental correlations are significantly different from 0 (p < 0.05).

^§^Phenotypic correlations are significantly different from 0 (p < 0.05).

A/E is the percentage of the phenotypic correlation that is caused by genes (A) or environment (E), based on the following equation: ${r}_{p}=(\sqrt{{h}_{BP}^{2}}\times {r}_{G}\times \sqrt{{h}_{HRV}^{2}})+(\sqrt{{e}_{BP}^{2}}\times {r}_{E}\times \sqrt{{e}_{HRV}^{2}})$.

**Table 4:** Bivariate quantitative genetic analyses of HRV, HR and BRS with office beat-to-beat resting SBP/DBP examining the genetic, environmental, and phenotypic correlations.

	N	Office beat-to-beat SBP				Office beat-to-beat DBP			
		Genetic correlation, r_G_(SE)	Environmental correlation, r_E_(SE)	Phenotypic correlation, r_P_	Proportions of r_P_ (A/E)	Genetic correlation, r_G_(SE)	Environmental correlation, r_E_(SE)	Phenotypic correlation, r_P_	Proportions of r_P_ (A/E)
lnSDNN	1155	0.06 (0.20)	−0.10^*^ (0.05)	−0.07^§^	0.12/0.88	−0.01 (0.20)	−0.07(0.05)	−0.06^§^	0.03/0.97
lnRMSSD	1155	−0.01 (0.19)	−0.14^*^ (0.05)	−0.12^§^	0.02/0.98	−0.09 (0.20)	−0.11^*^ (0.05)	−0.10^§^	0.15/0.85
lnHF	1126	0.20 (0.20)	−0.17^*^ (0.05)	−0.09^§^	0.23/0.77	0.17 (0.20)	−0.14^*^ (0.05)	−0.08^§^	0.22/0.78
lnLF	1126	0.16 (0.20)	−0.08(0.05)	−0.03	0.31/0.69	0.22 (0.21)	−0.07(0.05)	−0.02	0.40/0.60
lnVLF	1126	0.22 (0.22)	−0.08(0.05)	−0.03	0.35/0.65	0.19 (0.22)	−0.06(0.05)	−0.02	0.36/0.64
lnTP	1126	0.22 (0.21)	−0.12^*^ (0.05)	−0.06^§^	0.28/0.72	0.22 (0.22)	−0.10^*^ (0.05)	−0.05	0.30/0.70
lnHR	1155	0.29 (0.14)	0.17^*^ (0.05)	0.20^§^	0.40/0.60	0.39^†^ (0.14)	0.14^*^ (0.05)	0.21^§^	0.50/0.50
lnBRS	1124	−0.22(0.18)	−0.10 (0.05)	−0.13^§^	0.39/0.61	−0.17(0.19)	−0.08 (0.05)	−0.10^§^	0.36/0.64

Models were adjusted for age, sex, BMI, age^2^, age × sex, and age^2^ × sex.

SBP: systolic blood pressure; DBP: diastolic blood pressure; r_G:_ genetic correlation; r_E_: environmental correlation; r_P_: phenotypic correlation; SE: standard error; SDNN: standard deviation of normal-to-normal intervals; RMSSD: root mean square of successive differences; HF: high frequency; LF: low frequency; VLF: very low frequency; TP: total power; HR: heart rate; BRS: baroreceptor sensitivity.

^†^Genetic correlations are significantly different from 0 (p < 0.05).

*Environmental correlations are significantly different from 0 (p < 0.05).

^§^Phenotypic correlations are significantly different from 0 (p < 0.05).

A/E is the percentage of the phenotypic correlation that is caused by genes (A) or environment (E), based on the following equation: ${r}_{p}=(\sqrt{{h}_{BP}^{2}}\times {r}_{G}\times \sqrt{{h}_{HRV}^{2}})+(\sqrt{{e}_{BP}^{2}}\times {r}_{E}\times \sqrt{{e}_{HRV}^{2}})$.

HR had consistently positive, yet still weak phenotypic correlations with BP (r: 0.005 to 0.21). The correlation coefficients seemed higher with office beat-to-beat BP (Table 4) compared with ambulatory BP (Table 3) (SBP: 0.20 vs. 0.005 to 0.04 and DBP: 0.21 vs. 0.02 to 0.07) and environmental factors always significantly contributed to the phenotypic correlation of HR with office beat-to-beat SBP and DBP (p < 0.01).

BRS showed consistently negative and similar phenotypic correlations with daytime ambulatory BP (r: −0.09 to −0.08; Table 3), sleeping ambulatory BP (r: −0.08 to −0.07; Table 3), 24-hour ambulatory BP (r: −0.10 to −0.08; Table 3) and office beat-to-beat BP (r: −0.13 to −0.10) (Table 4).

## Discussion

The main purpose of our study was to estimate the heritability of BP and cardiac autonomic nervous system activity and explore whether shared genetic factors partly explained the inverse relationship. Our study echoed previous findings on the heritability estimates and small negative correlations between BP and indices of autonomic nervous system activity. However, we did not identify significant genetic contributions to those phenotypic correlations except for the correlations between HR and office beat-to-beat BP. Environment contributed more than genetic factors in our bivariate analyses, especially for the office beat-to-beat BP measurements.

Our study found moderate heritability for BP, which varied for different measurement conditions. It was higher for daytime or ambulatory BP measurements compared with sleeping or office beat-to-beat BP, respectively. These results were in line with the univariate BP heritability estimates we published previously,^15^ and similar to other family studies conducted in different populations (SBP: ranging from 0.30 to 0.37; DBP: ranging from 0.22 to 0.41)^23–25^. Some of this variation in BP heritability estimates may be due to different genes or sets of genes contributing to BP regulation in different measurement conditions such as office vs. real life or daytime vs. nighttime which was also shown in previous twin studies^26,27^. We also found differences in heritability estimates between HR and HRV indices, where HR (h^2^ = 0.32) had a higher heritability than all the HRV indices (h^2^ from 0.11 to 0.21), which were somewhat smaller than estimates in the Kibbutzim family study^28^. The heritability of BRS in our study of 21% was very similar to the estimates reported by Riese *et al*. (22%)^29^.

We found consistently negative phenotypic correlations between HRV and BP and positive ones between HR and BP measures. This pattern of relationships was consistent with both cross-sectional^3,4^ and prospective cohort studies^5,6^. The latter longitudinal studies even showed that low HRV, high HR or reduced BRS preceded the onset of HTN indicating that they may cause new onset HTN. An alternative explanation of such findings might still be that genetic factors causing low HRV overlap with those conferring increased risk for HTN. However, even the phenotypic correlation coefficients we observed were relatively small compared with well-known risk factors of HTN such as obesity.

Our study identified a significant genetic contribution to the correlation between HR and office beat-to-beat DBP, but did not find significant genetic correlations of HRV/BRS indices with either ambulatory or office beat-to-beat BP. Also, significant environmental correlations were found for various HRV indices (including SDNN, RMSSD, HF and TP) and HR with office beat-to-beat SBP or DBP, but hardly any with ambulatory BP. One possible reason may be that the ECG from which HRV measures were derived and beat-to-beat BP were measured simultaneously in supine position, while ambulatory BP was measured during the previous day. Thus, correlations with ambulatory BP measures could have been expected to be smaller. Compared with ambulatory BP, office beat-to-beat BP measurements may be better suited to identify the shared genetic and/or environmental components with autonomic nervous system and some common genes may co-contribute to the synchronous regulation of HR and office beat-to-beat BP.

A strength of this family study is that it was conducted in a homogeneous Arab population, in which the individuals had a very similar genetic background (tradition of encouraging consanguineous marriage) and shared environmental effects (living in a relatively isolated region). Consequently it was expected to have better power to examine the genetic contributions to BP and HRV related traits than other studies conducted in outbred, heterogeneous populations^13^. Another strength is that BP values were measured using 24-hour ambulatory monitoring, which is believed to better predict target organ damage than conventional BP methods^30^. Furthermore many efforts were made to ensure data quality: (1) the effects of hypertension medication were corrected to optimally preserve genetic variability as recommended by previous studies^16,17^; (2) we combined up-up and down-down measures in a single BRS variable and conducted sensitivity analysis for BRS with and without missing data to optimally utilize the available data, and; (3) HRV measurements were all determined following the standards recommended by the Task Force of the European Society of Cardiology and the North American Society of Pacing and Electrophysiology^7^. However, there were also some limitations of this study. Firstly, although our study contained data on more than 1200 participants, the sample size may still not be large enough. The associations we are investigating were small and some of them were not significantly statistically correlated. So, underlying genetic contributions to these correlations may also be too small to detect with the current sample size. Interestingly, using results from meta-analyses of genome-wide association studies of both HRV indices and BP based on very large sample sizes^31^ we recently did find significant negative genetic correlations between HRV and BP ranging between −0.35 and −0.20. Secondly, our study was conducted using a cross-sectional design and only major confounding factors, i.e. age, age^2^, sex, BMI and age-sex and age^2^-sex interactions. Additional covariates, including but not limited to central adiposity measures such as waist-to-hip ratio may have slightly changed the results.

To conclude, our study in a homogeneous Arab population echoed prior findings on HRV, HR and BRS’ effects on BP regulation. But it also indicated that those correlations may be relatively weak compared with other well-known risk factors. In addition, the genetic effects contributing to those correlations were not as significant as expected and only observed under some specific conditions (e.g. HR and office beat-to-beat BP) which indicated the complexity of underlying mechanisms explaining those correlations.

## Supplementary information

Heritability and genetic and environmental correlations of heart rate variability and baroreceptor reflex sensitivity with ambulatory and beat-to-beat blood pressure
