# Supplementary material for: Heritability and genetic and environmental correlations of heart rate variability and baroreceptor reflex sensitivity with ambulatory and beat-to-beat blood pressure
Source: Sci Rep. 2019 Feb 7;9:1664. doi: 10.1038/s41598-018-38324-6 (PMC6367510; doi:10.1038/s41598-018-38324-6)
Supplement: Supplementary file 1 — Heritability and genetic and environmental correlations of heart rate variability and baroreceptor reflex sensitivity with ambulatory and beat-to-beat blood pressure [file 41598_2018_38324_MOESM1_ESM.pdf]

Title page

Heritability and genetic and environmental correlations of heart rate variability and baroreceptor reflex sensitivity with ambulatory and beat-to-beat blood pressure

Tengfei Man[1], Harriëtte Riese[2], Deepali Jaju[3], M. Loretto Muñoz[1], Mohammed O Hassan[4], Said Al Yahyaee[5], Riad Bayoumi[6], Anthony G Comuzzie[7], John S. Floras[8], Arie M. van Roon[9], Ilja M. Nolte[1], Sulayma Albarwani[3+], Harold Snieder[1*+]

1. Department of Epidemiology, University of Groningen, University Medical Center Groningen, The Netherlands;

2. Interdisciplinary Center Psychopathology and Emotion regulation, Department of Psychiatry, University of Groningen, University Medical Center Groningen, The Netherlands;

3. College of Medicine and Health Sciences, Sultan Qaboos University, Muscat, Sultanate of Oman;

4. Canadian Health Centre, Muscat, Sultanate of Oman;

5.Department of Biochemistry, College of Medicine and Health Sciences, Sultan Qaboos University, Muscat 123, Sultanate of Oman;

6. College of Medicine, Mohammed Bin Rashid University for Medicine and Health Science, Dubai, UAE.

7. Department of Genetics, Texas Biomedical Research Institute, Texas, USA;

8. University Health Network and Mount Sinai Hospital Division of Cardiology, Department of Medicine, University of Toronto, Toronto, Ontario, Canada;

9. Department of Vascular Medicine, University of Groningen, University Medical Center Groningen, The Netherlands.

+ These authors contributed equally to this work

* Corresponding author:

H. Snieder, PhD
Department of Epidemiology
University Medical Center Groningen
Hanzeplein 1
PO Box 30001
9700 RB Groningen
The Netherlands
Tel +31 50 361 0887
Fax +31 50 361 4493
E-mail h.snieder@umcg.nl

This study was supported by a grant from His Majesty Sultan Qaboos Strategic Research Trust Fund (SR/MED/PHYS/04/01), Ministry of Health, Sultanate of Oman. The authors declare no conflict of interest.

Supplementary Materials

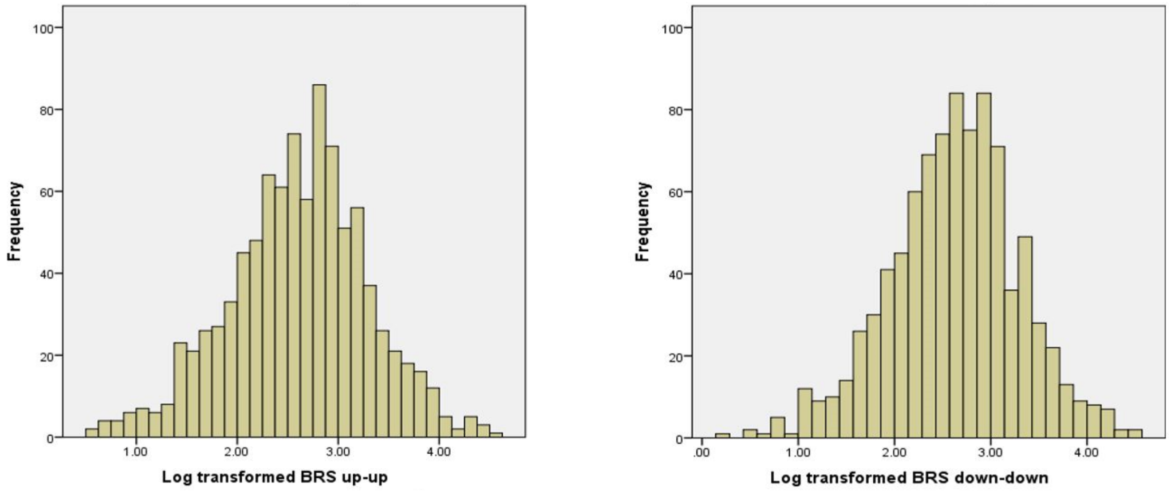

Figure 1 Histogram of the distribution of log transformed BRS up-up (left) and BRS down-down (right) variables

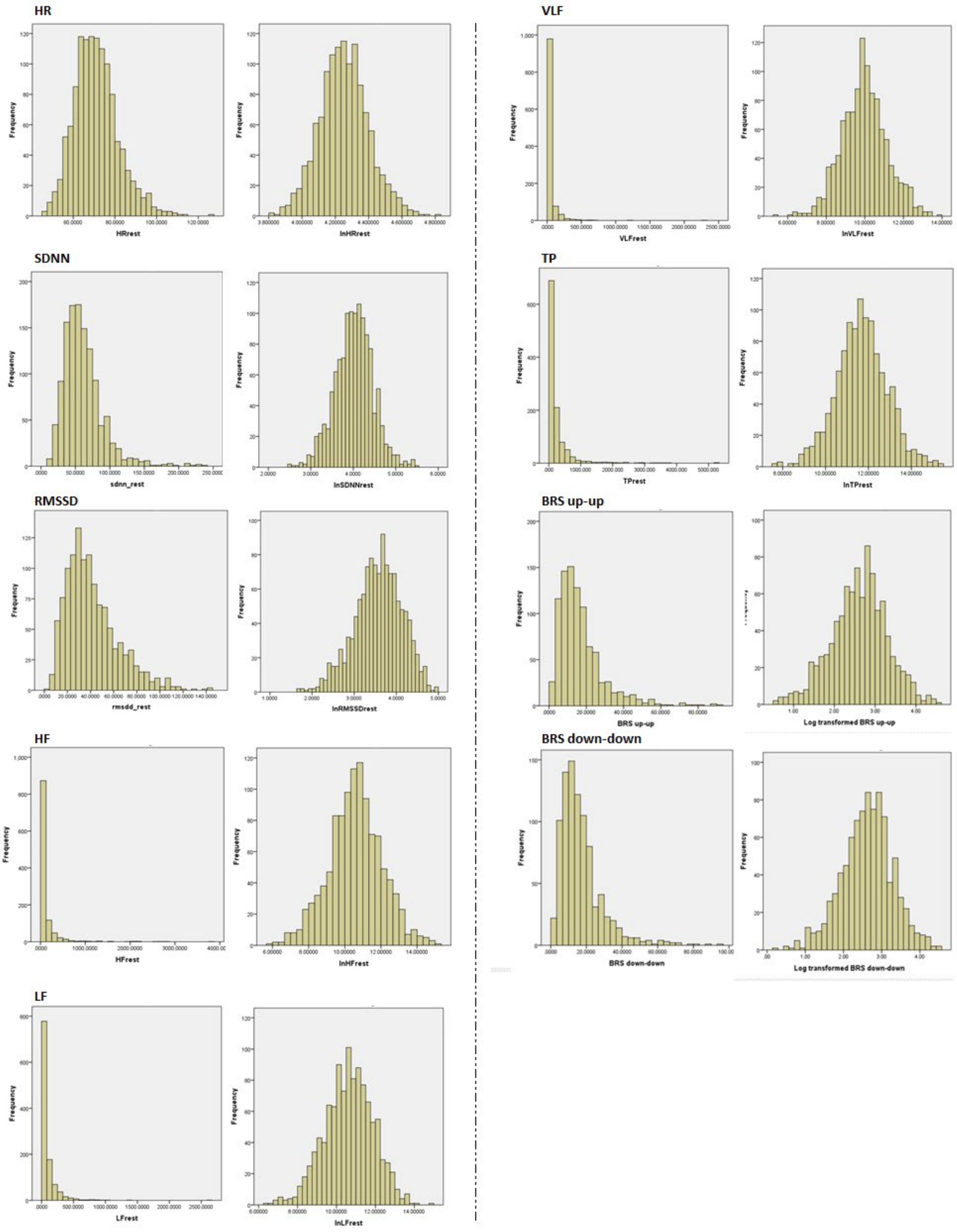

Figure 2 Distributions of HR, HRV (including SDNN, RMSSD, HF, LF, VLF, TP) and BRS (up-up and down-down) measurements prior (left) and after (right) natural log transformation
